# Population-based estimates of age-specific cumulative risk of breast cancer for pathogenic variants in *ATM*

**DOI:** 10.1186/s13058-022-01518-y

**Published:** 2022-04-01

**Authors:** Anne-Laure Renault, James G. Dowty, Jason A. Steen, Shuai Li, Ingrid M. Winship, Graham G. Giles, John L. Hopper, Melissa C. Southey, Tú Nguyen-Dumont

**Affiliations:** 1 Precision Medicine, School of Clinical Sciences at Monash Health, Monash University, Clayton, VIC 3168 Australia; 2 Centre for Epidemiology and Biostatistics, Melbourne School of Population and Global Health, The University of Melbourne, Parkville, VIC 3010 Australia; 3 Centre for Cancer Genetic Epidemiology, Department of Public Health and Primary Care, University of Cambridge, Cambridge, CB1 8RN UK; 4 Royal Melbourne Hospital, Parkville, VIC 3050 Australia; 5 Department of Medicine, The University of Melbourne, Parkville, VIC 3010 Australia; 6 Cancer Epidemiology Division, Cancer Council Victoria, Melbourne, VIC 3004 Australia; 7 Department of Clinical Pathology, Melbourne Medical School, The University of Melbourne, Parkville, VIC 3010 Australia

**Keywords:** *ATM*, Breast cancer predisposition, Genetic risk factors, Age-specific cumulative risk, Penetrance

## Abstract

**Background:**

Multigene panel tests for breast cancer predisposition routinely include *ATM* as it is now a well-established breast cancer predisposition gene.

**Methods:**

We included *ATM* in a multigene panel test applied to the Australian Breast Cancer Family Registry (ABCFR), a population-based case–control–family study of breast cancer, with the purpose of estimating the prevalence and penetrance of heterozygous *ATM* pathogenic variants from the family data, using segregation analysis.

**Results:**

The estimated breast cancer hazard ratio for carriers of pathogenic *ATM* variants in the ABCFR was 1.32 (95% confidence interval 0.45–3.87; *P* = 0.6). The estimated cumulative risk of breast cancer to age 80 years for heterozygous *ATM* pathogenic variant carriers was estimated to be 13% (95% CI 4.6–30).

**Conclusions:**

Although *ATM* has been definitively identified as a breast cancer predisposition gene, further evidence, such as variant-specific penetrance estimates, are needed to inform risk management strategies for carriers of pathogenic variants to increase the clinical utility of population testing of this gene.

**Supplementary Information:**

The online version contains supplementary material available at 10.1186/s13058-022-01518-y.

## Background

*Ataxia-Telangiectasia Mutated* (*ATM*) encodes a protein kinase involved in DNA damage repair. Bi-allelic pathogenic variants in *ATM* cause Ataxia–Telangiectasia (A–T), a complex phenotype with poor prognosis. Heterozygous carriers do not display the clinical features of A–T except for an increased predisposition to various cancers, including breast cancer. Women who meet genetic testing criteria due to a personal or family history of breast cancer and are heterozygous carriers of a pathogenic variant in *ATM* have been estimated to be at a two–fourfold increase in breast cancer risk compared to non-carriers [1, 2]. Several studies have reported breast cancer risk associated with carrying one missense pathogenic variant in *ATM* (c.7271T>G) to be high, setting it apart from other pathogenic variants in *ATM* in terms of the magnitude of associated breast cancer risk (e.g., OR 11.0 (1.42–85.7) *p* = 0.0019 [3]). Previously, using a population-based family study, we estimated the penetrance of *ATM* c.7271T>G to be 52% (95% CI 28–80%; HR = 8.6; 95% CI 3.9–18.9; *P* < 0.0001) [4]. Goldgar et al. estimated penetrance of likely pathogenic variants in *ATM* using data from 27 families (15 of which carried c.7271T > G) to be 60% to age 80 years [5].

Two recent large-scale, landmark studies have provided more insight into the prevalence of *ATM* pathogenic variants in population settings [6, 7]. In these studies, 0.6–0.7% of affected women who did not carry a pathogenic variant in *BRCA1* or *BRCA2* were found to carry a pathogenic variant in *ATM*. Dorling et al*.* estimated an odds ratio (OR) of breast cancer risk of 2.1 (95% confidence interval (CI) 1.35–3.23, *p* < 0.001) for *ATM* pathogenic variant carriers compared to non-carriers. Hu et al*.* estimated an OR of 1.8 (95% CI 1.46–2.27, *p* < 0.001) and, by combining this OR with the SEER breast cancer incidence rates for the population, derived an estimate of lifetime absolute risk of breast cancer greater than 20% for *ATM* pathogenic variant carriers. These studies have clearly established the relative risk of breast cancer associated with *ATM* pathogenic variants for women in the general population. However, for the purposes of genetic counselling, estimates of age-specific cumulative risks (penetrance) are more clinically useful yet are limited for *ATM* pathogenic variants*.*

We conducted a genetic screen of *ATM* in the Australian Breast Cancer Family Registry (ABCFR), an Australian population-based case–control–family study of breast cancer, with the purpose of estimating the prevalence and penetrance of *ATM* pathogenic variants in this cohort.

## Methods

### Study participants and genomic data generation

The ABCFR is a population based, case–control–family study of breast cancer, carried out in Australia (Melbourne and Sydney) as part of the international Breast Cancer Family Registry (BCFR). Case-probands were over-sampled for those with early-onset breast cancer, but were sampled irrespective of family history. Blood-derived germline DNA from 1480 case probands and 864 control probands were screened by targeted-sequencing of the coding regions and proximal intron–exon junctions of *BRCA1* (NM_007294.4), *BRCA2* (NM_000059.4) and *ATM* (NM_000051.4). Details of study participant characteristics and selection, sequencing and data processing and variant filtering and annotation methods have been published previously [8] and are summarized in Additional file 1: Fig. S1.

### Genetic variant selection

Our statistical analyses focused on rare pathogenic or predicted deleterious variants, hereafter refer to as “pathogenic”. Rare variants were defined as those identified in the non-Finnish European population of gnomAD [9] and in the ABCFR with a minor allele frequency (MAF) ≤ 0.001. To define pathogenic variants, ClinVar annotations of “Pathogenic” or “Likely Pathogenic” were used (accessed July 2021). Predicted deleterious variants included truncating variants that were not present in ClinVar and a subset of missense substitutions as described below.

For ATM*,* the specific domains in which missense substitutions have been more commonly associated with A-T are the FAT, kinase and FATC domains. Therefore, missense substitutions were scored using the web version of Align-GVGD [10], and our statistical analysis included missense substitutions that i) fell into the PFAM FAT (residues 2096–2849), PPI3_PI4_kinase (residues 2713–2962) and FATC (residues 3025–3056) domain definitions and ii) received an Align-GVGD grade of C55 or C65, indicating that they were evolutionary unlikely (deleterious).

### Statistical analyses

Hazard ratios (HRs) and age-specific cumulative risks (penetrance) were estimated as described in detail in [8]. Briefly, HRs for carriers of pathogenic *ATM* variants were estimated by segregation analysis as implemented in the statistical package MENDEL version 3.2, then the estimated cumulative risk to a given age was derived from the estimated HR. All estimates were appropriately adjusted for the population-based ascertainment of the families, and an unmeasured polygene was used to model any residual familial aggregation of breast cancer. Non-carrier incidences were chosen so that the average incidence for carriers and non-carriers (weighted by the carrier frequency) was the age-specific population incidence rates for Australia in the period 1998–2002, as obtained from Cancer Incidence in Five Continents [11]. The population cumulative risk to age 80 was taken to be 10.9%. The allele frequency of all pathogenic *ATM* variants combined was taken to be 0.001. All *p* values were 2-sided, and a *p* value threshold of 0.05 was used to define statistical significance.

## Results

Targeted-sequencing was successfully performed on the germline DNA of 1476/1480 (99.7%) case-probands and 861/864 (99.7%) control-probands. A pathogenic *ATM* variant was identified in 25/1476 (1.7%) of case-probands and 9/864 (1.0%) of control-probands (Table 1, Additional file 2: Table S1 provides ClinVar and Align-GVGD/domain information, Additional file 3: Table S2 provides baseline characteristics by carrier status). None of the probands were found to also carry a pathogenic variant in *BRCA1* or *BRCA2.*

**Table 1:** *ATM* variants identified by targeted-sequencing in the case-and control-probands participating in the Australian Breast Cancer Family Registry

	Variant type	HGVSc ^a^	HGVSp ^a^	Number of Relatives Who Are Carriers/Tested/Total	Number of Relatives with Breast Cancer Who Are Carriers/Tested/Total
Case proband	Nonsense	NM_000051.4:c.9139C>T	NP_000042.3:p.Arg3047*	2/2/31	0/0/1
	Nonsense	NM_000051.4:c.5623C>T	NP_000042.3:p.Arg1875*	0/0/16	0/0/0
	Nonsense	NM_000051.4:c.8098A>T	NP_000042.3:p.Lys2700*	0/1/17	0/0/0
	Nonsense	NM_000051.4:c.7792C>T	NP_000042.3:p.Arg2598*	0/0/22	0/0/0
	Nonsense	NM_000051.4:c.1396C>T	NP_000042.3:p.Gln466*	3/3/33	0/0/1
	Nonsense	NM_000051.4:c.5515C>T	NP_000042.3:p.Gln1839*	0/0/15	0/0/0
	Nonsense	NM_000051.4:c.8977C>T	NP_000042.3:p.Arg2993*	1/1/30	0/0/0
	Nonsense	NM_000051.4:c.3658G>T	NP_000042.3:p.Glu1220*	0/1/68	0/1/1
	Frameshift	NM_000051.4:c.5156delA	NP_000042.3:p.Asn1719Ilefs*5	2/2/19	0/0/0
	Frameshift	NM_000051.4:c.8264_8268delATAAG	NP_000042.3:p.Tyr2755Cysfs*12	1/1/90	1/1/1
	Frameshift	NM_000051.4:c.1355delC	NP_000042.3:p.Thr452Asnfs*21	0/1/17	0/0/0
	Frameshift	NM_000051.4:c.5712dupA	NP_000042.3:p.Ser1905Ilefs*25	0/0/23	0/0/1
	Frameshift	NM_000051.4:c.3802delG	NP_000042.3:p.Val1268*	0/1/20	0/0/0
	Frameshift	NM_000051.4:c.7957_7960dupATTA	NP_000042.3:p.Thr2654Asnfs*3	2/2/15	0/0/0
	Frameshift	NM_000051.4:c.6671dupT	NP_000042.3:p.Met2224Ilefs*25	0/3/53	0/0/1
	Splice region	NM_000051.4:c.8418+5_8418+8delGTGA		0/1/36	0/0/1
	Splice region	NM_000051.4:c.8418 + 5_8418+8delGTGA		0/2/36	0/0/1
	Splice acceptor	NM_000051.4:c.8672-6_8672-2delCTTTA		0/0/22	0/0/0
	Splice acceptor	NM_000051.4:c.1236-2_1237delinsTTTTT		0/0/46	0/0/0
	Missense	NM_000051.4:c.8122G>A	NP_000042.3:p.Asp2708Asn	3/6/83	0/0/3
	Missense	NM_000051.4:c.8494C>T	NP_000042.3:p.Arg2832Cys	0/0/19	0/0/0
	Missense	NM_000051.4:c.7271T>G	NP_000042.3:p.Val2424Gly	2/4/19	2/2/2
	Missense	NM_000051.4:c.8494C>T	NP_000042.3:p.Arg2832Cys	0/0/18	0/0/0
	Missense	NM_000051.4:c.8741T>C	NP_000042.3:p.Ile2914Thr	1/2/34	0/0/0
	Missense	NM_000051.4:c.8494C>T	NP_000042.3:p.Arg2832Cys	0/0/31	0/0/0
Control proband	Nonsense	NM_000051.4:c.9151G> T	NP_000042.3:p.Gly3051*	0/0/28	0/0/2
	Nonsense	NM_000051.4:c.1039G> T	NP_000042.3:p.Glu347*	0/0/16	0/0/0
	Nonsense	NM_000051.4:c.64G>T	NP_000042.3:p.Glu22*	0/0/25	0/0/0
	Nonsense	NM_000051.4:c.5029G>T	NP_000042.3:p.Glu1677*	0/0/33	0/0/2
	Splice acceptor	NM_000051.4:c.3078-1G>A		0/0/23	0/0/0
	Missense	NM_000051.4:c.8734A>G	NP_000042.3:p.Arg2912Gly	0/0/19	0/0/0
	Missense	NM_000051.4:c.7375C>T	NP_000042.3:p.Arg2459Cys	0/0/13	0/0/0
	Missense	NM_000051.4:c.8558C>T	NP_000042.3:p.Thr2853Met	0/0/36	0/0/2
	Inframe deletion	NM_000051.4:c.7638_7646delTAGAATTTC	NP_000042.3:p.Arg2547_Ser2549del	0/0/23	0/0/0

^a^Variant nomenclature according to the Human Genome Variation Society (HGVS), HGVS.c for coding DNA and HGVS.p for protein variants, based on transcript sequence NM_000051.4, +1 as A of ATG start codon; * denotes a termination codon as per the HGVS nomenclature

The risk estimates were based on 1029 relatives of the 34 probands who carried a pathogenic *ATM* variant. Of these relatives, 33 had germline DNA for testing, and 19 were female breast cancer cases. In addition, a number of relatives had cancers of other anatomical sites (though only breast cancer contributed to our analyses): 20 lung, 20 prostate, 12 colorectum, 8 stomach and 56 at other anatomical sites (none reported more than five times). The relatives included 17 known carriers and 16 known non-carriers of the pathogenic *ATM* variant identified in the proband, though ungenotyped people also contributed to our estimates via their phenotypes and their relationships to genotyped people.

**Fig. 1:**
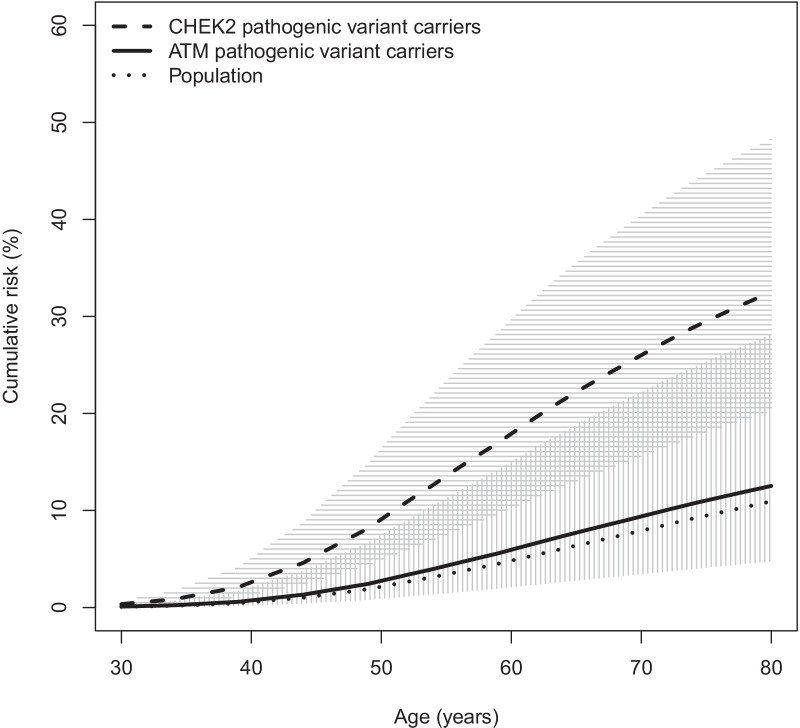
Average age-specific cumulative risk (penetrance) of breast cancer, for Australian women (dotted line) and for female carriers of pathogenic *ATM* variants combined (solid line) and pathogenic *CHEK2* variants combined (dashed line), with confidence intervals for carriers (grey region)

The estimated breast cancer HR for carriers of pathogenic *ATM* variants in the ABCFR was 1.32 (95% CI 0.45–3.87; *P* = 0.6). Excluding the rare missense variants that are predicted to be deleterious but do not yet have a ClinVar classification (Additional Table 1) did not change the HR for carriers (1.36 (95% CI 0.44–4.16; *P* = 0.6). Based on the above HR estimate for all pathogenic *ATM* variants combined, cumulative risks for these carriers to various ages were calculated (Fig. 1, Additional file 4: Table S3). Carriers had a 13% (95% CI 4.6–30) probability of developing breast cancer by the age of 80 years.

## Discussion

Variant classification remains a critical challenge to fully realize the clinical utility of genetic testing for *ATM*. This important issue and others have been identified as areas of priority by the *International Consortium on ATM and Cancer*, initiated in 2019, which brings together researchers and clinicians who aim to use a collaborative, multidisciplinary approach to addressing key questions about the cancer risks for carriers of a pathogenic *ATM* variant [12].

For *ATM*, as is the case for most breast cancer predisposition genes, truncating variants are, with a few exceptions, predicted to lead to loss of protein function and are classified as pathogenic. However, focusing on the FAT, kinase and FATC domains in ATM, Tavtigian et al*.* reported that the risk associated with carrying missense variants identified in these three domains (in aggregate) could be higher than that of protein truncating variants (in aggregate) [13]. Only a handful of *ATM* missense variants have been reported to be pathogenic in ClinVar. Missense variants represent a large proportion of the rare variants identified in our study: 70/129 (54%) of all rare variants in our study were missense substitutions but only 3/70 are classified as pathogenic in ClinVar.

We previously calculated cumulative risk estimates for *CHEK2* in the ABCFR and observed that the penetrance estimates for pathogenic variants in *CHEK2* and *ATM* are not statistically different (Fig. 1) [8]. There is an urgent and currently unmet need to provide robust information that can inform risk management strategies for carriers of pathogenic variants in intermediate risk genes such as *ATM* and *CHEK2,* as these genes are now routinely included on gene panels for cancer predisposition.

While these two genes are considered *bona fide* breast cancer predisposition genes, national best practice recommendations are only emerging to guide the management of women found to carry pathogenic variants in these genes. This situation results in a feeling of uncertainty and anxiety in these women [14].

## Conclusion

Further international collaboration is required, potentially via the newly formed *International Consortium on ATM and Cancer* [12]*,* to refine the penetrance estimates, identify relevant modifying factors (including the polygenic risk score), and the risk of other cancers for carriers of *ATM* pathogenic variant carriers.

## Supplementary Information

**Additional file 1**. **Figure S1** Overview of study workflow.

**Additional file 2**. **Table S1** Characteristics of the *ATM* variants identified by targeted-sequencing in the Australian Breast Cancer Family Registry. Legend: HGVSc and HGVSp: variant nomenclature according to the Human Genome Variation Society (HGVS), HGVS.c for coding DNA and HGVS.p for protein variants. ClinVar: classification accessed July 2021. NA indicates that classification was not available. AGVGD: for missense variants only, grade obtained from the web version of Align-GVGD. Only C55 and C65 scores were considered deleterious in this analysis. ATM domain: PFAM FAT (residues 2096–2849), PPI3_PI4_kinase (residues 2713–2962) and FATC (residues 3025–3056) domain definitions were used (missense variants only).

**Additional file 3**. **Table S2** Baseline characteristics of *ATM* pathogenic variant carriers, by carrier status.

**Additional file 4**. **Table S3** Cumulative risks of breast cancer to various ages for carriers of *ATM* pathogenic variants.

## Data Availability

The dataset supporting the conclusions of this article is included within the article. used and/or analyzed during the current study are available from the corresponding author upon reasonable request.

## Abbreviations

ABCFR: Australian Breast Cancer Family Registry
Align-GVGD: Align Grantham Variation Grantham Deviation
A-T: Ataxia-Telangiectasia
CI: Confidence interval
HR: Hazard ratio
MAF: Minor allele frequency
