# Supplementary figures and images for: Population-based estimates of age-specific cumulative risk of breast cancer for pathogenic variants in ATM
Source: Breast Cancer Res. 2022 Apr 1;24:24. doi: 10.1186/s13058-022-01518-y (PMC8973562; doi:10.1186/s13058-022-01518-y)

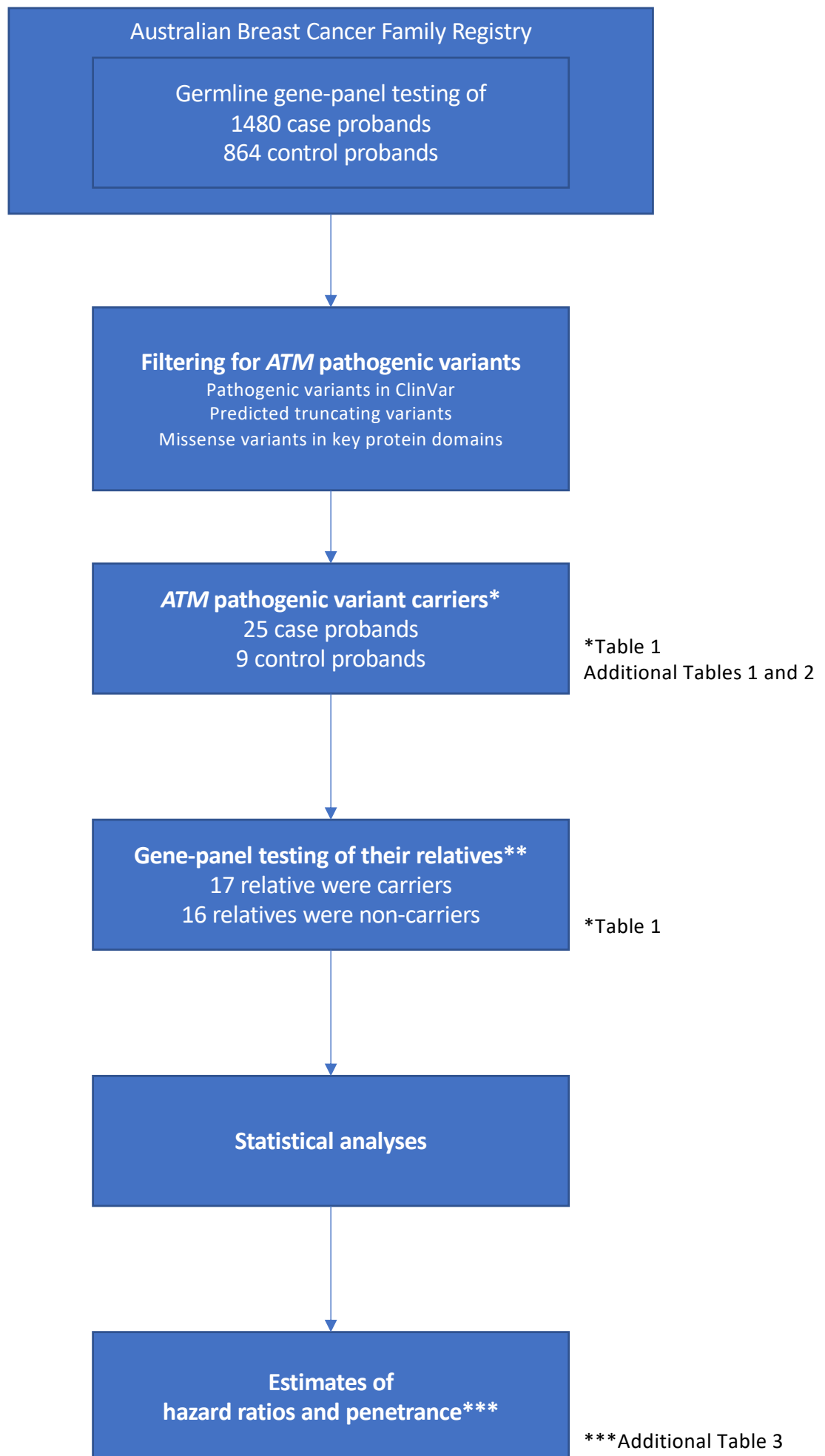

Supplement: Supplementary file 1 — Additional file 1. Figure S1 Overview of study workflow. [file 13058_2022_1518_MOESM1_ESM.pdf]
